# New York Odontological Society

**Published:** 1894-07

**Authors:** 

**Affiliations:** New York Odontological Society


### Reports of Society Meetings.

### NEW YORK ODONTOLOGICAL SOCIETY.

A REGULAR meeting of the New York Odontological Society was held on Tuesday evening, April 17, 1894, at the New York Academy of Medicine, No. 17 West Forty-third Street, New York City, Dr. Brockway presiding.

The minutes of the previous meeting were read and approved.

### INCIDENTS OF OFFICE PRACTICE AND CASUAL COMMUNICATIONS.

*The President.*—I notice many gentlemen present who are not members, and we extend to them a cordial invitation to take part in the discussions.

*Dr. S. E. Davenport.*—It pleases me to call attention to the fact that Dr. Lord has recently designed some very valuable instruments for removing tartar from the teeth. He has already presented these instruments to us, and has in private received the commendation of many gentlemen who have used them. I wish to add my thanks, and say they are useful to me almost every day. Some little time ago I asked his permission to make some modification in the shape of the instruments, because I was unable, with the forms designed by him, and my methods of manipulation, to reach the roots of the molars satisfactorily. Dr. Lord's instrument-maker has made a number of instruments according to my suggestion, retaining the valuable feature of the Lord sickle-scaler,—the groove in both upper and lower edges. The modification which I suggested is merely a double curve, the instruments being rights and lefts, which enables me to operate upon the molars without stretching the lips of the patient very much; and I will pass around several pairs for examination.

*Dr. Lord.*—No one need to feel that they must ask permission to change the curves or angles of the instruments; of course, every one would prefer to suggest his own curves. The groove in the edges of the hook or curve of the instrument is the only feature in which it differs from the old (so-called) sickle instrument.

*Dr. Littig.*—I desire to speak of the way in which I utilize old amalgam. I take the scraps, melt them up, and pour into an ingot, which, of course, drives off all the mercury. We frequently want the form of a tooth to make a band or clasp upon, that will be stronger than plaster; and when I have taken my impression, and I realize that I want such a tooth in metal, I simply mix up this old amalgam which I have filed up, and pack it into my impression, making the cast of a small tooth, on which I can make my band or clasp. You may ask, "Why don't you use Melotte's fusible metal?" but we do not always have it at hand, and I can use this with wax or any impression material. I file it up after I have melted it, with a coarse rasp. I have with me some of the teeth that I have already made.

*Dr. Jackson.*—I have here a model, with an appliance for retaining loose teeth, which I brought this evening on account of some remarks that were made at the February meeting regarding methods of holding incisors that had become loose from pyorrhœa or other cause. I would like to present the apparatus, although I propose at some future time to describe it more fully.

Dr. Pain suggested a method of anchoring a gold bar to the cuspid teeth which passed on the lingual side of the incisors, from which gold fingers extended forward to clasp the loose teeth.

I have constructed a crib appliance for holding a tooth that had fallen out from pyorrhœa, and I could see from Dr. Pain's remarks that the same style apparatus could be readily used for retaining loose teeth. The appliance is made of German silver, although gold or platinum-iridium can be utilized with equal advantage.

A small wire bar is passed on the lingual side of the teeth, near the gum, and anchored, with crib attachments, to a bicuspid on each side. Wires are arranged at the junction of the teeth to be retained. They should be soldered to the bar and extend to the cutting-edge of the incisors, where they should pass over to the labial side, which forms a hook. It may be necessary, in some cases, to grind the upper side of the wire flat, and cut a slight notch, so that the wire shall rest level with the cutting-edge; or the inner part of the hook portion of the wire can be flattened and the notches between the teeth made to correspond.

*Dr. Francis.*—I would like to ask a question in regard to oxyphosphate of zinc. From the experience of the gentlemen present, which do they prefer? I formerly thought Weston's was pretty good, but in the last year I think it has failed very much.

*The President.*—I hope the gentlemen will answer that question satisfactorily. I do not know of any one who is entirely satisfied with the preparation of oxyphosphate of zinc that he is using. If there is any one present who can answer that, I hope he will do so.

*Dr. Lord.*—I have used Hammond's oxyphosphate with the most satisfaction. The liquid does not crystallize. It maintains its clearness to the end of the bottle, and apparently is as good as when first opened.

*Dr. Bogue.*—What about its durability?

*Dr. Lord.*—I have only used it about a year, but, as far as I have noticed it, it wears well.

*Dr. Bogue.*—I have a lot in the mouth that is six or seven years old. It is Poulson's. It can be mixed so it will wear away in six months, or it can be made sometimes to last six years. All I know about it I learned from Dr. Arthur. He told me not to use oxyphosphate that had a liquid, but to use such preparations as require the melting of the crystals of the phosphoric acid at the time of using. He recommended this one of Poulson's, and I will tell you how I use it. Lay your powder out on a cold slab, or glass. Please put emphasis on the "cold," as I have had gentlemen tell me to warm it slightly. I keep my glass in the refrigerator. The oxide of zinc is put on the glass; the phosphoric acid is melted on a bit of platinum over the spirit-lamp, at so low a degree that I hold the platinum in my fingers. It is kept over the flame until it is thoroughly melted, and is then immediately poured upon the glass. Of course, it becomes chilled at once. My instruments are ready, the rubber dam is on, and a cushion of chamois-skin, slightly oiled, is ready and fixed to my table. Before I begin to mix, all these things are prepared. I mix it as stiff as it can be made. I take it off the glass and knead it with my fingers on my way to the cavity, and when I get there it has softened a second time. Then it is packed in as rapidly as possible, sometimes with a matrix, sometimes not. It is as stiff as hard putty to begin with, and when I am through it is very stiff. When it has become hard, it is whittled down with a tiny sickle-shaped lancet, and when finally polished down with fine tapes,—polished slowly, so as not to heat it,—then it is dried off with a current of air, and stearine or paraffine is melted on the surface. I have some fillings that have been in a long time. One is in the mouth of my daughter. It was to stay there temporarily, but it has now been in over six years, and has not deteriorated. I am pleased to bring this Poulson's material again before my brethren. It takes a little more time and a great deal more care. One wants to know the preparation before he begins to put it in the mouth, lest it set too quickly; but when you are careful the best results can be had with it that I have been able to get from any of the phosphate cements.

*The President.*—What is the object of the oiled chamois-skin?

*Dr. Bogue.*—To run my burnisher quickly over this pad, so it does not stick to the phosphate as I am packing it in.

*The President.*—Are you particular to mix it slowly?

*Dr. Bogue.*—No; I add it as quickly as I can thoroughly.

*The President.*—Is that not contrary to the usual advice in the directions which come with the phosphates?

*Dr. Bogue.*—That I do not know. I have not read the directions for some time. Of course, you cannot mix thoroughly and mix with very great rapidity.

*Dr. Ives.*—I never saw but one oxyphosphate filling that was really successful. It was made with Fletcher's, I believe, and the fluid was very opaque. It was a large corono-approximal cavity in a lower molar. It had been in for fifteen years, and I could not see the least possible waste in any shape or manner.

*The President.*—That is one of the most favorable places for an oxyphosphate filling, I believe.

*Dr. Ives.*—Yes, it is.

*Dr. Bogue.*—And yet Fletcher, in Ash's Catalogue, in response to the offer, I think, of one hundred pounds on the part of the British Odontological Society for experiments to be made in that direction, indulges in some merriment at the expense of the Society, saying he had spent at least fifteen thousand pounds, and knew there was a fortune in store for any one who could discover such an oxyphosphate.

*Dr. Davenport.*—Regarding the durability of Poulson's zinc phosphate in the mouth, I have known the material, with the greatest possible care in insertion, and with all conditions as favorable as described by Dr. Bogue, to fail as quickly in certain mouths as any zinc phosphate one could select with failure in view; but I think such cases are exceptional, and that it is really the most reliable of our cements. I have used very satisfactorily, as I presume all have in approximal cavities, gutta-percha at the cervical wall and for perhaps one-quarter the depth of the cavity, preferably with the matrix, completing the filling with Poulson's or other phosphates. It seems to me that if we do fill approximal cavities with zinc phosphate, the gutta-percha at the gum margin will certainly add to the durability of the filling.

*Dr. Stockton.*—My experience is very much like that of the other gentlemen present. As Dr. Davenport has just said, sometimes one cement does well and at other times it does badly. I think I have used them all.

I used, at one time, Dr. Welch's oxyphosphate, and thought of that when the doctor spoke of the filling which had been in such a long time. One filling, I remember, lasted about seven years. The same material used in many other cases failed. There is a great difference in this material; some hardens at once and others take a long time. I find that Britton's cement, manufactured in Trenton, New Jersey, is quite a good one. I have used it lately. In almost all approximal cases, as Dr. Davenport has said, I never trust the oxyphosphate without placing in gutta-percha at the cervical wall. I might say that gutta-percha does even better than oxyphosphate in many cases. I often wonder that we succeed as well as we do with the materials we have.

*Dr. Bogue.*—Is it ever proper to destroy the pulp in the treatment of calcic abscess? If so, why? If not, why not?

It has been generally supposed that dental caries occurs either in the enamel (white decay), or in the dentine after the enamel has been penetrated, but that practically it never occurs in the cementum.

I present this evening a typical case,—one of perhaps a dozen or more that have come into my hands within the past four or five years,—where the decay is distinctly in the cementum, and has been progressing so long that the pulp became exposed.

The position of the cavity is so unusual that it seems to have escaped the notice of three different dentists, all of whom have treated the case differently, and now that it has come into the hands of a fourth practitioner, a course has been adopted quite at variance with any of the usual ones.

The conditions were as follows: The patient, a clergyman of over fifty years of age; the three right superior molars all loose, all extremely sensitive to heat and cold, growing worse week by week until the effort to masticate is so painful that mastication is given up on that side. Then the brush becomes a source of pain, and is used with "great care." (I quote the words of the patient, which I interpret to mean "great care" not to hurt himself, but not great care to keep the tartar off.) After some weeks (eight or ten) of suffering, no relief being found, the second molar was extracted, as an abscess had begun to form at its root. This loss interfered with vocalization, making it difficult to pronounce certain words, and wearisome in case of long-continued speaking. The sensitiveness of the other two teeth continued unabated, and they seemed to be getting continually looser.

At this period he came into my hands.

After getting the history of the case and making an examination, I diagnosed as follows:

*First.*—Calcic abscesses on all three of the teeth, owing to the deposition of tartar and the consequent recession of the gums.

*Second.*—The probable death of the pulp of the second molar from that recession, causing the abscess which precipitated its loss.

*Third.*—The certainty that the pulp of the wisdom-tooth would speedily follow in the course of that of the second molar, and die spontaneously.

*Fourth.*—That the pulp of the first molar was causing pain from a slight exposure through the cavity of decay in the cementum.

*Treatment.*—With a fine sharp drill I opened at once into the pulp-chamber of both first and third molars, and applied arsenic.

At this point I interrupt my written paper to allow an opportunity for discussion, questioning, and criticism, which must necessarily arise when so many of us are considering the same case

*Dr. Davenport.*—Many years ago I gave up having any respect for the pulp in a tooth which was so uncomfortable that it could not be brushed or used in mastication. It seems to me that it would have been impossible for Dr. Bogue to adopt any other course than to destroy the life and, consequently, the extreme sensitiveness, of teeth in the condition described, his duty being to give the patient comfort and the use of the teeth.

*Dr. Ives.*—What else could he possibly do, the pulp being exposed, than to destroy it?

*Dr. Bogue.*—The pulp in the wisdom-tooth was not exposed, although the first-molar pulp was. This is not the first time I have destroyed the pulp because the patient was urging me to extract the tooth. One lady complained that it gave her so much pain she thought the tooth was worth less than her comfort. I did destroy the pulp, and I think the tooth is there yet, after some eighteen years. On several occasions, notwithstanding the injunction that we are not to do so, I have destroyed the pulp with a view of keeping the tooth longer in the mouth. I certainly supposed this treatment would be attacked. I should be glad to have it done, because after the attacking party has ventilated its ideas, and I mine, we might get at the facts.

*Dr. Nash.*—I have a case somewhat in point. A gentleman of the dental profession called upon me one Sunday morning, in great pain, having a lower molar with a large open cavity, but no pulp exposure. I applied some creosote and oil of cloves in the cavity thinking it might afford relief, and in the course of two or three hours he came back. The pain was intolerable, and I resorted to heroic measures by taking up the engine-drill and deliberately exposing the pulp, liberating congested blood; I then applied my creosote and oil of cloves with some satisfaction. He said he was relieved from that moment. Several days afterwards I made an application of arsenic. He had intense pain that night, but since then he has been free from any discomfort; and as soon as opportunity offers, I shall proceed in the usual manner to fill the tooth permanently.

*Dr. Brewster.*—May I ask Dr. Bogue, how long after the extraction of the second molar was the impression taken?

*Dr. Bogue.*—I do not know; about two or three weeks. I did not see the patient until after the second molar had been extracted.

*Dr. Brewster.*—Then there was not much inflammation at the time it came to you?

*Dr. Bogue.*—No. I would say now that the patient came back and reported that during twenty-four or forty-eight hours he suffered considerable pain from the application I made in the cavity in the first molar. Be it understood that the arsenic I put in was intermingled with the cotton preparation, so that it was seemingly dry. I packed it into that root canal so as to be sure that nothing touched the gum. I drilled into the wisdom-tooth right through the anterior face, and packed the arsenicated cotton in there. He suffered twenty-four hours with that first molar, and after fortyeight hours he went to his regular dentist at home (another city than New York), and that gentleman removed the arsenic from the first molar, but did not touch the wisdom-tooth. The pain in the wisdom-tooth ceased on the second day, and he said he had been perfectly comfortable since, and the tooth had become firm in its socket. The pain in the first molar was continuing, and the tooth was looser than ever. I drilled in and removed what pulp I could from the first molar, and treated it with vinaigre de Pennés, which is a preparation made of benzoic acid, boracic acid, benzoin, carbolic acid, and various other things of that sort. I have most of the ingredients, but not all. It is a proprietary medicine in France. Its antiseptic qualities are such that, about four months ago, I threw a few drops upon a piece of beefsteak that was not very fresh, and another piece that was fresh, and I have kept them in my office ever since. Its use has been vaunted in a curious way, namely, by using it after the application of arsenic, and without the extraction of the pulp in wisdom-teeth, especially where pain has been present from exposure or partial exposure. The cavities are then supposed to be filled up with anything you like, and the patient never afterwards complains.

*A Member.*—Is not that something like the Herbst method?

*Dr. Bogue.*—Herbst's method is conceived without knowledge of the conditions involved. Herbst is a good mechanic, but not a physician nor an anatomist, still less a pathologist, as I understand it. The other man is. In this case, the third molar was rendered comfortable. The gentleman can brush his tooth again. The first molar has its posterior buccal root denuded of periosteum to the apex, the palatine root half-way, and the anterior buccal pretty well up. In these cases, after the arsenic has done its work, the tartar may be removed, the patient may again eat on that tooth, and the tooth may be restored to fairly good condition. I have seen them remain for years, when the patient had begged to have them extracted previously to that application.

Before I sit down I want to present before the Society two teeth which one of our members has just handed me. One of them seems to completely upset what I stated here a month ago. Here is a tin-foil bottom to a gold filling. The amount of tin in proportion to the gold is very small, but the mixed filling has been there eight years. So reads the label. So far as I can see, it is in pretty good order. I will take it home and make a more careful examination. If it is in good condition, it is the first one I have seen. As has been stated, there is a great difference between putting gold among tin and putting tin among gold. It seems as though this were a case in which the tin had lasted.

Here is a case that the doctor was kind enough to show, much like the cases that I have been describing, in which a tooth with a living root has had tartar secretions clear up to the other root. It was extremely sensitive to heat and cold. The posterior root was evidently devitalized.

Dr. Francis then read a paper on the subject of cohesiveness of tin fillings. Dr. Francis also read a short editorial by Dr. Barrett, of Buffalo, entitled "The Virtues of Tin," in the *Dental Practitioner*.

(For Dr. Francis's paper, see page 443.)

DISCUSSION.

*Dr. Ives.*—If I remember rightly, in Dr. Francis's first paper, no one took issue with him that tin was not cohesive. As far as my remarks were concerned, I said that it was not cohesive in the same sense that gold was; that it was what we call mechanical cohesion, —that you could not lay two pieces of tin together and get the same results as when you lay together two pieces of gold. All these gentlemen claim that the surfaces must be fresh and perfectly pure. Thinking of the paper after the last meeting, I made a cavity in a natural tooth, and with one single instrument, and hand-pressure entirely, I built out a contour filling. I have it here with me. The last three layers I dipped in water, and, under the same kind of pressure, they stayed there. What I meant in my remarks at the last meeting was that tin-foil could not be used in the same sense that gold-foil was used, and that I had never seen it done. The foil in this filling has been lying in my drawer for eight or ten years. It was mechanical cohesion in every sense of the word.

*Dr. Lord.*—I do not think that any one at the last meeting said that tin was not cohesive in some sense.

When Dr. Francis used the word cohesive as applied to tin, I said Dr. Francis certainly does not mean that tin is cohesive in the sense that cohesive gold is cohesive, or as we use the term in speaking of cohesive gold. That was all that I said in regard to the cohesiveness of tin, just then. Later, I said in my little paper that it was mechanical cohesion, as I understand it. The folds or layers are forced into each other with suitable points, and in that way we get cohesion, and in the same way we get cohesion of soft gold, and so get a solid mass. The tin being softer, it can be made the more solid of the two, but the gold will wear the longest when exposed to or in a masticating surface, as it is a harder metal.

*Dr. Nash.*—I would suggest that one of these contour fillings that have been made in the teeth out of the mouth be taken from the cavity, so as to see exactly the form of cohesion that has taken place there. I have experimented, since hearing Dr. Francis read his paper, to a limited extent only, with some of Mr. Kearsing's tin, and I succeeded in making a contour filling and getting a good hard surface. I afterwards removed that filling, and concluded that the cohesive properties of tin are not such as we expect in using gold.

*Dr. Lord.*—I think it is a mistake to say that the tin must be fresh, or freshly made, to work well. I am using tin that was made, as I stated at the last meeting, over twenty years ago. It is just as bright, can be packed as solid, and works in every way as well as when it was first made. I notice no difference, as I stated at that time.

*Dr. Bogue.*—Dr. Ives, I believe, is willing to take out that tin filling, and show us what cohesion has taken place. It seems to me that we are a little at variance, because, possibly, we do not all understand in the same way. Suppose I take a quart of plaster and mix it with water and let it set, I have a fairly solid mass; but if I mix with that plaster a quart of sand, while I have a solid mass I have not the same kind of solidity. My friend, Dr. Lord, may say that his old tin-foil works just as well as fresh foil. I grant that it does, so far as the sensitiveness of our fingers can guide us; but if we were to examine them, we would find in the old foil filling atoms that had been oxidized. It could not be otherwise. Put the foil where you please, and there will be oxidation on the surface of the tin, while there would not be on gold.

*Dr. Stockton.*—I do not think it matters much whether tin is cohesive or not. I have no doubt that it is. All metals are more or less so. What we want to know is, Is tin better than other materials, or as good, for filling teeth? We don't care so much about cohesion as whether tin saves teeth or not. For approximal cavities, and for filling the cervical walls, where you will finish with gold, I say it is one of the best materials that can be used. We do not care to bother about cohesion, because it would take three or four times as long to fill a tooth by that process as it would by the English or Scotch process of "stuffing," as they call it. You can fill a tooth three or four times quicker by the wedging process than you can by cohesion. Use tin just as you do soft gold-foil. I remember Dr. Darby, at the Columbian Congress, spoke on the subject, and has since written upon it, and I suppose everybody now will take up tin. We had copper amalgam some time ago, and we all wish we had never used it; but we won't wish that about tin, because it has stood the test. It is very useful in children's teeth especially. Not very many years ago we discussed the good qualities of tin at societies all over the country, and it has come back again. I think the conservative practice is to use tin where we think it best, gold where that is best, gutta-percha and oxyphosphate where we consider they are the best. They all have their uses.

*Dr. Bogue.*—Some months ago I brought before this Society a photograph, which you possibly may remember. It was that of a young girl, twenty-one years of age, a good portion of whose jaw was amputated, and a substitute was screwed on to the two ends of the bone by Dr. Michaels, of Paris. Since then, under the direction of Dr. Péon, he has performed another operation, which is to construct the shoulder-joint of the left shoulder of rubber and platinum, and screw that fast to the humerus below and the scapula above. I have with me pictures of the apparatus in place, one with the arm open, one of the apparatus in place again in a different position, and one of the young man himself.

I saw him some time after the operation, and saw him use his arm, which he did a little awkwardly. He said he could use his fork, but not easily. A similar thing has been performed in Lyons, and Dr. Abbé, on Forty-ninth Street, New York City, has inserted a glass tube into the artery of a dog and trotted him out, still alive and pretty well. The day before yesterday, I saw the Paris edition of the New York *Herald*, containing this young man's portrait and two of the photographs that I have shown you, with a description of the apparatus. As it was done by one of our own specialty, Dr. Michaels, I take great pleasure in bringing it before the Society. It seems to me that to him a great deal of credit is due for a marvellous operation. His invention of the shoulder-joint is clear cut. It was a very simple affair, resembling almost a ball-and-socket joint; only the ball, instead of being in the socket in this way [illustrating], has platinum around the outside, so it moves in both directions.

*Dr. Littig.*—How long ago was that operation performed?

*Dr. Bogue.*—A year ago, and the patient's condition has since improved in every way.

Adjourned.

John I. Hart, D.D.S.,
*Editor New York Odontological Society.*

